# First Report of an Extensively Drug-Resistant ST23 *Klebsiella pneumoniae* of Capsular Serotype K1 Co-Producing CTX-M-15, OXA-48 and ArmA in Spain

**DOI:** 10.3390/antibiotics10020157

**Published:** 2021-02-04

**Authors:** Marta Hernández, Luis López-Urrutia, David Abad, Mónica De Frutos Serna, Alain A. Ocampo-Sosa, José María Eiros

**Affiliations:** 1 Laboratorio de Biología Molecular y Microbiología, Instituto Tecnológico Agrario de Castilla y León (ITACyL), 47071 Valladolid, Spain; hernandez.marta@gmail.com (M.H.); AbaGarDa@itacyl.es (D.A.); 2 Servicio de Microbiología, Hospital Universitario del Río Hortega, 47012 Valladolid, Spain; llopezu@saludcastillayleon.es (L.L.-U.); mdefrutossernad@saludcastillayleon.es (M.D.F.S.); jmeiros@saludcastillayleon.es (J.M.E.); 3 Laboratorio de Microbiología Clínica y Molecular, Instituto de Investigación Sanitaria Marqués de Valdecilla (IDIVAL), 39011 Santander, Spain

**Keywords:** *Klebsiella pneumoniae*, OXA-48, CTX-M-15, ArmA, hybrid virulence/resistance plasmids, extensively drug-resistant, hypervirulent *Klebsiella pneumoniae*

## Abstract

An extensively drug-resistant (XDR) *Klebsiella pneumoniae* isolate MS3802 from a tracheostomy exudate was whole-genome sequenced using MiSeq and Oxford Nanopore MinION platforms in order to identify the antimicrobial resistance and virulence determinates and their genomic context. Isolate MS3802 belonged to the clone ST23 and presented a capsular serotype K1, associated with hypervirulent *K. pneumoniae* (hvKp) isolates. The isolate harboured a chromosomally encoded *bla*_CTX-M-15_ gene and contained a large IncHI1B hybrid virulence/resistance plasmid carrying another copy of the *bla*_CTX-M-15_ and the virulence factors *iucABCD-iutA, iroBCDN, rmpA* and *rmpA2.* The carbapenemase gene *bla*_OXA-48_ was found in a Tn*1999*-like transposon and the 16S rRNA methylase *armA* gen located in the vicinity of other antibiotic-resistant genes on an IncM2 plasmid. This study represents, to the best of our knowledge, the first description of a *bla*_CTX-M-15_-, *bla*_OXA-48_- and *armA*-harbouring *K. pneumoniae* of ST23 and capsular serotype K1 in Spain. Our report emphasizes the importance of implementing new surveillance strategies to monitor the risk of emergence and spread of such XDR and hypervirulent *K. pneumoniae* isolates.

## 1. Introduction

*Klebsiella pneumoniae* is one of the most significant opportunistic human pathogens. This Gram-negative bacterium is responsible for hospital- and community-acquired infections such as urinary tract infections, pneumonia and severe bacteremia, associated with high morbidity and mortality rates [1]. A new “hypervirulent” *K. pneumoniae* (hvKp), which differed from the classical *K. pneumoniae* (cKp) by showing a hypermucoviscous phenotype, was described for the first time more than 20 years ago in Taiwan [2]. Since then several new cases have been reported in different regions of Europe and America, but the most important outbreaks have occurred in Asian countries [3]. In our country, some hvKp have also been documented [4,5,6]. hvKp strains can cause severe infections, such as pyogenic liver abscesses (PLA), osteomyelitis, endophthalmitis and metastatic meningitis in healthy individuals [7]. The hypermucoviscous/hypervirulent phenotype is generally associated with the *magA* (*wzy*-K1) gene, present in the gene cluster for K1 capsular polysaccharide [7,8], and/or *rmpA*, the regulator of mucoid phenotype A, which activates capsule production and may be located in the chromosome or a large virulence plasmid of both hvKp K1 and K2 capsular phenotypes [9,10]. However, not all hvKp strains show a hypermucoviscous phenotype as defined by a positive string test [11], nor the presence of the K1 or K2 capsule by itself can be used to define a strain as hvKp [12]. On the other hand, there are several other virulence factors associated with hypervirulent K1 and K2 phenotypes such as the siderophores aerobactin and yersiniabactin, the iron uptake operon, *kfu*, the fimbrial gene cluster, *kpc*, and *allS*, related to allantoin metabolism, just to mention a few [13].

At least 134 capsular serotypes have been described so far [14], but most of the hvKp strains belong to serotype K1, followed to a lesser extent by serotypes K2, K5 and K57 [13,15]. According to multilocus sequence typing (MLST) and core genome MLST (cgMLST) analyses, the majority of hvKp strains of serotype K1 are confined within the clonal complex CC23 that includes sequence types ST23, ST26, ST57 and ST1633, which are associated with specific virulence factors that confer the hypervirulent phenotype and perhaps also an increased bacterial fitness. While, hvKp strains of serotype K2 can be found in a variety of unrelated sequence types, including ST25, ST65, ST66 and ST86, and are genetically more diverse [13,15,16,17].

Generally, hvKp are highly susceptible to most commonly used antimicrobial agents, with the exception of ampicillin [18]. However, this scenario seems to be changing over time with the global spread of genetic mobile elements and conjugative plasmid carrying several antibiotic resistance determinants [15]. Multidrug-resistant hvKp (MDR-hvKp), including extended-spectrum β-lactamases (ESBL)-producing isolates and carbapenem-resistant hvKp (CR-hvKp), have been described recently [19,20,21,22,23,24,25,26]. MDR and/or carbapenem-resistant cKp (CR-cKp) isolates have also the capacity to acquire virulence plasmids such as pLVPK [27] or pK2044 [28], thus becoming hvKp [29]. 

In this study, we reported the first isolation of an XDR-*K. pneumoniae* isolate of sequence type 23 (ST23) and capsular serotype K1 co-producing OXA-48, CTX-M-15 and ArmA from a tracheostomy exudate sample collected in a Spanish hospital.

## 2. Results and Discussion

### 2.1. Isolation and Phenotypic Tests of K. pneumoniae MS3802

The MS3802 isolate was originally recovered from a tracheostomy exudate sample from a male patient who was hospitalized due to injuries caused by a motor vehicle accident. The isolate was identified as *K. pneumoniae* by using both a Vitek-2 instrument and matrix-assisted laser desorption ionization—time of flight mass spectrometry (MALDI-TOF MS). The MS3802 isolate was classified as XDR according to standard definitions for acquired resistance [30]. MS3802 was non-susceptible to most of the antibiotics tested, remaining only sensitive to imipenem, meropenem, doripenem and tetracycline (Table 1). Management of hvKp infections is more difficult due to the appearance of MDR or XDR phenotypes. However, in spite of being XDR, isolates like MS3802 that still remain susceptible to few antibiotics may be treated at least with a carbapenem. MS3802 did not show a hypermucoviscous phenotype, as the string test was negative (colonies did not stretch more than 5 mm using a standard bacteriological loop) and was considered as mucoid. A hypermucoviscous phenotype is usually associated with hvKp. However, not all the hvKp isolates are positive to the string test. For that reason, hypervirulence must be defined by the presence of certain virulence factors such as aerobactin, yersiniabactin and the *rmpA/rmpA2* genes associated with this phenotype, as well as the clinical manifestations of the disease [12]. Rapid identification of *K. pneumoniae* capsular serotypes K1 and K2 usually associated with hvKp may also be carried out by immunochromatographic strip assays [31] or PCR [23].

### 2.2. Whole-Genome Sequencing of the XDR K. pneumoniae MS3802

**Figure 1:**
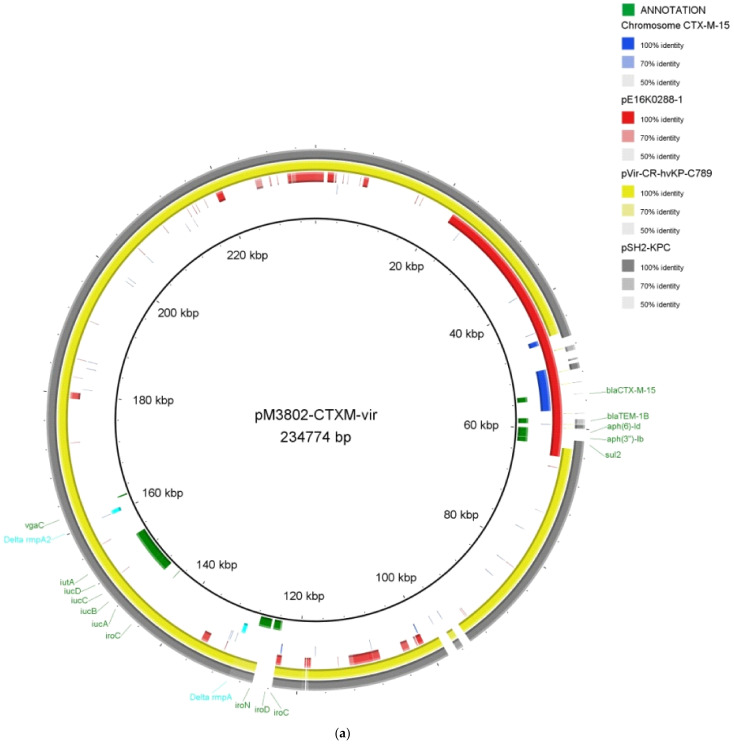

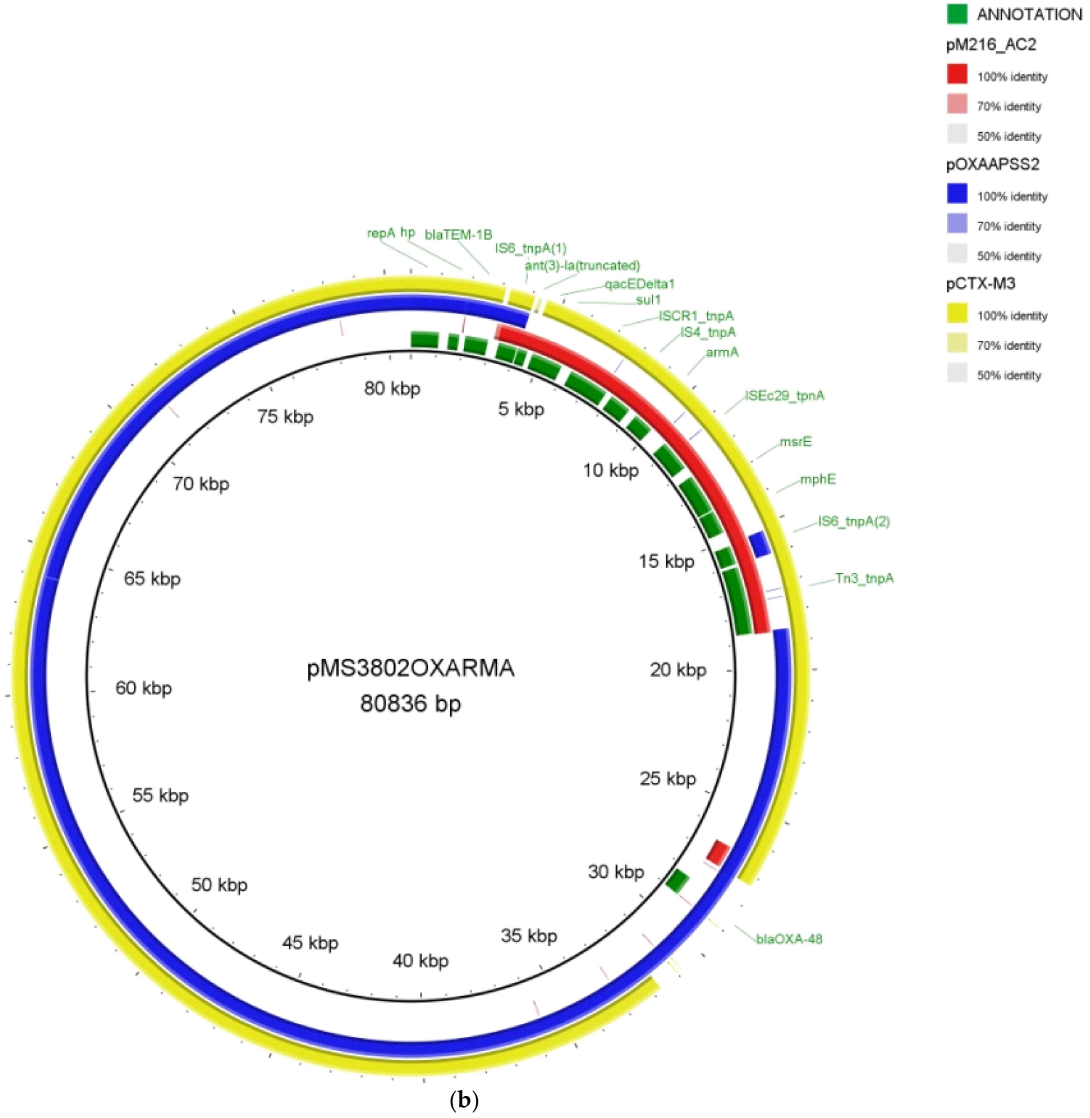
Blast Image Generator (BRIG) analysis of the hybrid virulence/resistance *bla*_CTX-M-15_-carrying plasmid pMS3802-CTX-vir (**a**) and plasmid pMS3802OXARMA containing the *bla*_OXA-48_ and *armA* genes (**b**) found in the XDR *K. pneumoniae* isolate MS3802. Comparative analysis of plasmids pMS3802-CTX-vir and pMS3802OXARMA with closely related plasmids using the BLAST Ring Image Generator is shown in each case. The concentric rings display similarity between the reference sequence in the inner ring and the other sequences in the outer rings. The different colour levels indicate a BLASTn result with a matched degree of shared regions, as shown to the right of each plasmid rings.

In order to know in detail the genetic basis of virulence and antimicrobial resistance, the MS3802 genome was fully sequenced. The isolate was confirmed to be *K. pneumoniae* of capsular serotype K1 and sequence type 23 (ST23) from the well-known hypervirulent clonal complex CC23. MS3002 has a circular chromosome of 5,933,527 bp. It also harbours two plasmids of 234,774 bp (pMS3802-CTXM-vir) and 80,836 bp (pMS3802OXARMA) (Figure 1). The genome GC% content of MS3802 was 56.95, and 5,717 protein-coding genes were predicted. According to the Virulence Factor Database (VFDB) search, the MS3802 isolate presented 114 virulence-related genes (Table S1). Among these were the chromosomally-encoded yersiniabactin system *irp1*, *irp2*, *ybtAEPQSTUX* and its receptor *fyuA*, the *wcaGHIJ* genes related with capsule biosynthesis, the *magA/wzy_K* gene coding for the mucoviscosity-associated protein, *allABCD* genes and regulators *allS* and *allR* for allantoin metabolism and the type 3 fimbrial genes *mrkABCDFHIJ*, all of them usually linked with hypervirulent clones such as ST23. 

**Figure 2:**
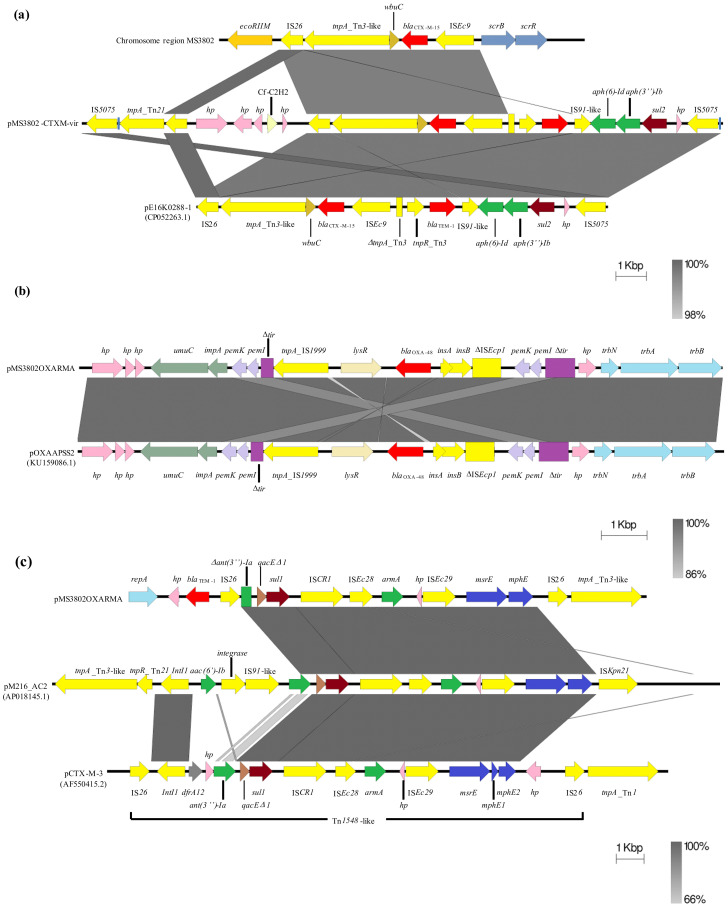
Schematic representation of the genetic location of the *bla*_CTX-M-15_ (**a**), *bla*_OXA-48_ (**b**) and *armA* (**c**) genes found in the *K. pneumoniae* isolate MS3802. The respective regions from MS3802 (chromosome or plasmid-encoded) containing *bla*_CTX-M-15_, *bla*_OXA-48_ and *armA* are compared with their closest homologous regions present in plasmids pE16K0288-1(CP052263.1), pOXAAPSS2 (KU159086.1) and pM216_AC2 (AP018145.1) and pCTX-M-3 (AF550415.2). Predicted open reading frames (ORFs) are represented on each lane by coloured arrows, with arrowheads showing the direction of transcription. Genes are highlighted according to similar functions: resistance to beta-lactams (red), aminoglycosides (green), sulfonamides (brown), macrolides (dark blue), mobile genetic elements (MGEs) or gene mobilization-related genes (yellow); ORFs with unknown (hp) functions are in pink, and those with other related functions have the same colour. Nonfunctional ORFs (deleted or disrupted) are represented by boxes. Homologous regions generated by a BLASTn comparison (≥99% identity) are represented as gray blocks connected across the strands. Colour shadings indicate the degree of similarity: the more saturated a similarity block, the more conserved are two ORF pairs or DNA regions. Figures were created using the Easyfig software [41].

Search for antimicrobial-resistant genes using Resfinder Database identified the presence of the *bla*_CTX-M-15_ gene flanked by the cupin fold metalloprotein *wbuC* gene, a Tn***3**-like*, IS*6* and IS*Ec9* transposase coding genes, which are frequently associated with CTX-M-15 (Figure 2A). Genes *qnrB1*, *ant(3’’)-Ia*, *dfrA14*, *fosA*, *bla*_SHV-190_ and the multidrug efflux system *oqxAB* were also present in the chromosome of MS3802. 

Other relevant antimicrobial-resistant determinants and virulence factors were found to be plasmid-encoded. The larger plasmid, pMS3802-CTXM-vir, belonged to the incompatibility group IncHI1B, and it is an example of hybrid resistance/virulence plasmid. Hybrid plasmids containing both drug-resistance and virulence-related genes have been recently described [23,32,33,34,35]. pMS3802-CTXM-vir carried a second copy of the *bla*_CTX-M-15_ gene within the same gene arrangement as the one located in the chromosome IS*26*-Tn*3*-*wbuC*-*bla*_CTX-M-15_-IS*Ec9* (Figure 2A), indicating that *bla*_CTX-M-15_ was acquired as a result of activities of these mobile genetic elements (MGEs). In particular, IS*Ec9* (also called ISEcp1) has been shown to be involved in mobilization and dissemination of CTX-M-like enzymes worldwide [36]. The *bla*_CTX-M-15_ gene, together with other antibiotic resistance genes (*bla*_TEM-1_, *aph(6)-Id*, *aph(3’’)-Ib* and *sul2*), was part of a large antibiotic resistance module (ARM) of 20,782 bp that was bracketed by two copies of the insertion sequence IS*5075* inserted in the same orientation with respect to each other (Figure 2A). Within this AMR was also a smaller internal module of 5408 bp flanked by two IS*26* elements containing five ORFs with unknown functions (hypothetical proteins) and a C2H2-type zinc finger protein. This module was inserted between a Tn*3*-like and a Tn*21* transposase coding genes (Figure 2A) and was likely acquired through a transposition process. A 13,626 bp fragment from the ARM containing the *bla*_CTX-M-15_ gene shared a 99.6% similarity with the backbone of plasmid pE16K0288-1 (GenBank accession number: CP052263.1). Identical direct repeat sequences (ATGGTCACTCCC) were found upstream of each IS*5075* copies suggesting that a possible transposition event occurred and led us to identify a new putative composite transposon. One of the IS*5075* copies was situated 81 bp upstream of a gene coding for a Tn*21*-like transposase, which is consistent with what has been previously observed which is that IS*5075* targets the terminal inverted repeats of the Tn*21*-like transposons of the Tn*3* family [37]. 

Plasmid pMS3802-CTXM-vir also encoded some virulence-related genes such as the ferric aerobactin *iucABCD* and aerobactin receptor *iutA,* the salmochelin siderophore *iroBCDN* and truncated copies of genes encoding the capsular polysaccharide synthesis (CPS) regulators *rmpA* and *rmpA2* (Figure 1A). According to previous studies, deletions of either *rmpA* or *rmpA2* genes resulted in a significant decrease of CPS [9,10,38]. The presence of nonfunctional alleles of these genes might be the cause for the absence of the hypermucoviscous phenotype in isolate MS3802. Nevertheless, the occurrence of *rmpA*/*A2* genes in K1 and K2 strains and their effect on CPS have been demonstrated to be dependent on the genetic background of each strain [10]. 

The smaller plasmid found in isolate MS3802, pMS3802OXARMA was an IncM2 type that shared a 99.8% similarity with the backbone of another IncM2 type plasmid, pOXAAPSS2 (GenBank accession number: KU159086.1) (Figure 1B). pMS3802OXARMA contained the carbapenemase gene *bla*_OXA-48_ carried by a Tn*1999*-like transposon inserted into the *tir* gene, encoding a plasmid transfer inhibition protein (Figure 2B). A previous study suggested that disruption of *tir* was associated with higher conjugation frequencies of *bla*_OXA-48_-bearing plasmids [39]. pMS3802OXARMA was also a carrier of the 16 S rRNA methylase *armA* that confers high resistance levels to aminoglycosides. The *armA* gen was located adjacent to an ORF coding an unknown protein and the MGEs IS*Ec28*, IS*Ec29* and IS*CR1*. The *msrE* and *mphE* macrolide resistance genes were found downstream of the IS*Ec29* element. Upstream of IS*CR1* were detected the genes *sul1*, *qacEΔ1* and a truncated *ant(3’’)-Ia* gene (Figure 2C). All these antimicrobial-resistant determinants were located within a putative class I transposon bounded by two copies of IS*26*. Downstream of the IS*26* positioned on the right side of the putative transposon was a Tn*3*-transposase gene. The *bla*_TEM-1_ gene was found upstream of the left side IS*26* copy. This gene arrangement was homologous to a Tn*1548*-like transposon and a gene cluster of 10,733 bp, located on IncM2 and IncA/C_2_ type plasmids, pCTX-M-3 (GenBank accession number: AF550415.2) and pM216_AC2 (GenBank accession number: AP018145.1), respectively (Figure 2C). This suggests that plasmid pMS3802OXARMA might have acquired this transposon-like structure from a closely related plasmid to pCTX-M-3 or pM216_AC2 *via* an IS*26*-mediated gene transfer and that association of *armA* and other resistance determinants with IS*26* found in this gene array could facilitate their introduction into new genetic locations. Both replicative and conservative transposition mediated by IS*26*-like elements has been demonstrated to be one of the major mechanisms of transmission of antimicrobial resistance determinants [40].

### 2.3. Demonstration of Transferability of MS3802 Plasmids

Conjugation experiments performed with sodium azide *Escherichia coli* J53 (Az^r^) as a recipient strain demonstrated the transferability of the plasmid pMS3802OXARMA at frequencies of 6.9 × 10^−4^ and 3.7 × 10^−4^ (transconjugants *per* donor) when J53 transconjugants were selected with piperacillin+sodium azide and amikacin+sodium azide, respectively. Transconjugants were selected with two different antibiotics as it was not known whether *bla*_OXA-48,_ *bla*_CTX-M-15_ and *armA* genes were located in the same plasmid. Antimicrobial susceptibility testing demonstrated that transconjugants exhibited resistance to amoxicillin/clavulanate, piperacillin/tazobactam, gentamicin, tobramycin, amikacin, netilmicin and showed intermediate-resistance to ceftazidime but remained susceptible to the rest of the agents tested (Table 1). PCR using specific primers [42,43,44] confirmed the presence of the *bla*_OXA-48_ and *armA* genes, but not the *bla*_CTX-M-15_ in the transconjugants, indicating that conjugation of plasmid pMS3802-CTXM-vir did not take place under the experimental conditions employed. Previous studies showed that hybrid virulence/resistance plasmids could not be transferred to *E. coli* by conjugation [32,45]. 

## 3. Conclusions

Our findings demonstrated the presence of a new IncHI1B hybrid drug-resistant virulent plasmid bearing the *bla*_CTX-M-15_ gene as well as a new IncM2 conjugative plasmid harbouring *bla*_OXA-48,_ *armA* and several other antibiotic-resistant determinants, thus mediating multidrug resistance in *K. pneumoniae*. To the best of our knowledge, this is the first case of an XDR *K. pneumoniae* of serotype K1 and ST23 from CC23, usually associated with hypervirulence, co-producing CTX-M-15, OXA-48 and ArmA in Spain. Although reports of multidrug carbapenemase-producing hypervirulent *K. pneumoniae* have so far been rare in our country, the occurrence of such isolates is particularly worrisome due to the confluence of both hypervirulence and multidrug resistance. This represents a new challenge clinicians and microbiologists have to overcome as it is a serious threat to public health. In spite of the existence of several studies about hvKp, there is still a paucity of information about multidrug-resistant hvKp, especially those multidrug-resistance strains carrying hybrid virulence/resistant plasmids. Definition of hypervirulence also requires further studies as virulence-related genes associated with hvKp strains are potential targets for new antibiotic treatments. On the other hand, the development of new effective diagnostic methods for the identification of MDR hvKp is urgently needed, as well as enhanced surveillance programs for the detection of such strains should be implemented. More prospective studies are also necessary in order to detect and determine the incidence of multidrug-resistant hvKp isolates in our hospital settings.

## 4. Materials and Methods

### 4.1. Strain Identification and Antimicrobial Susceptibility Testing

The isolate MS3802 was collected from a tracheostomy exudate from a male patient who was involved in a serious motor vehicle accident. Bacterial species identification was performed by using a Vitek-2 instrument (BioMérieux, Marcy l’Etoile, France), and subsequent confirmation was carried out by matrix-assisted laser desorption ionization–time of flight mass spectrometry (MALDI-TOF/Vitek-MS with SARAMIS MS-IVD v2.0) and by whole-genome sequencing (WGS).

The minimum inhibitory concentration of 27 antimicrobial agents was determined by broth microdilution according to the Clinical and Laboratory Standards Institute (CLSI) guidelines [46]. The EUCAST 2020 susceptibility breakpoints were applied (www.eucast.org/clinical_breakpoints/), except for cefoxitin, doripenem, netilmicin, minocyclin and tetracyclin, for which CLSI breakpoints were employed. *Pseudomonas aeruginosa* ATCC 27853, *Escherichia coli* ATCC 25,922 and *Klebsiella quasipneumoniae* subsp. *similipneumoniae* ATCC 700,603 were used as control strains for antimicrobial susceptibility testing.

### 4.2. String Test

In order to see if isolate MS3802 showed a hypermucoviscous phenotype, the string test was performed as described before [11]. A disposable inoculation loop was used to stretch away a bacterial colony grown on the agar plate. The formation of a viscous string >5 mm in length is defined as string test-positive, otherwise, the isolate was classified as mucoid.

### 4.3. Whole-Genome Sequencing and Bioinformatics Analysis

Genomic DNA from *K. pneumoniae* MS3802 isolate was purified with the DNeasy Blood & Tissue Kit (Qiagen) and sequenced by using both Illumina MiSeq instrument using reagents kit v3 for 2 × 300 paired-end libraries (Illumina, San Diego, CA, USA) as previously described [47] and Oxford Nanopore MinION (Oxford Nanopore Technologies (ONT), UK). Raw reads from the sequencing platform were directly analyzed by using an in-house bioinformatics pipeline [48] and the genome of *K. pneumoniae* subsp. *pneumoniae* ATCC 700721/MGH 78,578 as a reference strain. The options used in this study included quality control and filtering of the reads [49,50,51], genome assembly with SPAdes [52] and Quast [53], genome annotation with Prokka [54] and multi-locus sequence typing (MLST, T. Seemann, https://github.com/tseemann/mLst). Search of antibiotic resistance genes, virulence genes and plasmid replicons was done using BLAST [55] and ABRicate (T. Seemann https://github.com/tseemann/abricate) against ResFinder [56], Virulence Factor Database (VFDB) [57] and PlasmidFinder [58] databases, respectively. Point mutation investigation was performed with PointFinder [59]. Pangenome was created with Roary [60] and FastTree [61]. Serotyping was performed using Kaptive software [62].

### 4.4. Conjugation Experiments

The transferability of plasmids was investigated by carrying out conjugation experiments. *K. pneumoniae* MS3802 was used as a donor and *E. coli* J53, resistant to sodium azide (Az^r^), was used as the recipient strain. Transconjugants were selected on MacConkey agar plates supplemented with piperacillin (50 µg/mL) or amikacin (40 µg/mL) and sodium azide (100 µg/mL). Transconjugants were confirmed by PCR with specific primers for amplification of *bla*_OXA-48_, and CTX-M-encoding genes [42] and *armA* [43], as well as susceptibility testing (Table 1).

### 4.5. Nucleotide Accession Numbers

The whole-genome sequencing reads and annotated assembly of *K. pneumoniae* MS3802 and plasmids pMS3802-CTXM-vir and pMS3802OXARMA are available under the BioProject ID PRJNA674482 and GenBank accession numbers W222482 and MW222483, respectively. 

## Figures and Tables

**Table 1:** Antimicrobial susceptibility profiles of the *Klebsiella pneumoniae* isolate MS3802 and *Escherichia coli* J53 transconjugants with the plasmid pMS3802OXARMA.

Antimicrobial Agent	MIC (μg/mL)			
	*K. pneumoniae* MS3802	*E. coli* J53-Pip^r^	*E. coli* J53-AK^r^	*E. coli* J53 (wt)
Ampicillin	>128 (R)	>128	>128 (R)	64 (R)
Ampicillin/Sulbactam	>64 (R)	>64	>64 (R)	64 (R)
Amoxicillin/Clavulanic acid	>128 (R)	>128 (R)	>128 (R)	8 (S)
Aztreonam	>128 (R)	<0.125 (S)	<0.125 (S)	0.125 (S)
Piperacillin/tazobactam	>128 (R)	128 (R)	128 (R)	2 (S)
Cefuroxime	>64 (R)	8 (S)	8 (S)	8 (S)
Cefoxitin ^(*)^	16 (I)	8 (S)	8 (S)	2 (S)
Cefotaxime	>128 (R)	0.5 (S)	0.5 (S)	0.125 (S)
Ceftazdime	64 (R)	2 (I)	4 (I)	0.5 (S)
Cefepime	8 (R)	0.25 (S)	0.25 (S)	<0.06 (S)
Ertapenem	4 (R)	0.25 (S)	0.25 (S)	<0.06 (S)
Imipenem	2 (S)	0.5 (S)	0.5 (S)	0.125 (S)
Meropenem	1 (S)	0.5 (S)	0.5 (S)	<0.06 (S)
Doripenem ^(*)^	1 (S)	0.5 (S)	0.5 (S)	<0.06 (S)
Gentamicin	128 (R)	128 (R)	128 (R)	0.5 (S)
Tobramycin	128 (R	128 (R)	128 (R)	0.125 (S)
Amikacin	>128 (R)	>128 (R)	>128 (R)	0.125 (S)
Netilmicin ^(*)^	>128 (R)	128 (R)	>128 (R)	0.125 (S)
Ciprofloxacin	4 (R)	<0.06 (S)	<0.06 (S)	<0.06 (S)
Levofloxacin	8 (R)	<0.06 (S)	<0.06 (S)	<0.06 (S)
Tigecycline	2 (R)	0.25 (S)	0.125 (S)	<0.06 (S)
Tetracycline ^(*)^	1 (S)	0.5 (S)	1 (S)	0.5 (S)
Minocycline ^(*)^	8 (I)	4 (S)	2 (S)	4 (S)
Colistin	16 (R)	0.125 (S)	<0.06 (S)	<0.06 (S)
Trimethoprim/sulfamethoxazole	>128 (R)	0.125 (S)	0.125 (S)	<0.06 (S)
Cloramphenicol	32 (R)	8 (S)	8 (S)	8 (S)
Fosfomycin	>256 (R)	8 (S)	8 (S)	8 (S)

Pip^r^: *E. coli* J53 transconjugant selected in MHA supplemented with piperacillin and sodium azide, AK^r^: *E. coli* J53 transconjugant selected in MHA supplemented with amikacin and sodium azide. wt: wild-type strain. R: resistant, I: intermediate, S: susceptible. ^(*)^ Clinical and Laboratory Standards Institute (CLSI) breakpoints were applied for these antibiotics.

## Data Availability

The data presented in this study regarding the information about both the virulence and antimicrobial resistant genes are in Supplementary Table S1. Genome and plasmid sequences are available in https://www.ncbi.nlm.nih.gov/bioproject/ under the BioProject ID PRJNA674482 and GenBank accession numbers W222482 and MW222483, respectively.

## Supplementary Materials

The following are available online at https://www.mdpi.com/2079-6382/10/2/157/s1, Table S1: Virulence and antimicrobial resistance genes of isolate MS3802.
