# Supplementary material for: First Report of an Extensively Drug-Resistant ST23 Klebsiella pneumoniae of Capsular Serotype K1 Co-Producing CTX-M-15, OXA-48 and ArmA in Spain
Source: Antibiotics (Basel). 2021 Feb 4;10(2):157. doi: 10.3390/antibiotics10020157 (PMC7913926; doi:10.3390/antibiotics10020157)
Supplement: Supplementary file 1 [file antibiotics-10-00157-s001.zip › antibiotics-1092581-supplementary.pdf]

Gene	Gaps	Coverage	Identity	Accession
acrA	0/0	100.0	100.0	YP_002918166
acrB	0/0	100.0	100.0	YP_002918165
allA	0/0	100.0	100.0	YP_002918200
allB	0/0	100.0	100.0	YP_002918207
allC	0/0	100.0	99.9	YP_002918211
allD	0/0	100.0	100.0	YP_002918212
allR	0/0	100.0	100.0	YP_002918201
allS	0/0	100.0	100.0	YP_002918199
clbA	0/0	100.0	100.0	YP_006635473
clbB	0/0	100.0	100.0	YP_006635474
clbC	0/0	100.0	100.0	YP_006635475
clbD	0/0	100.0	100.0	YP_006635476
clbE	0/0	100.0	99.6	YP_006635477
clbF	0/0	100.0	100.0	YP_006635478
clbG	0/0	100.0	100.0	YP_006635479
clbH	0/0	100.0	100.0	YP_006635480
clbI	0/0	100.0	100.0	YP_006635481
clbL	0/0	100.0	100.0	YP_006635484
clbM	0/0	100.0	99.9	YP_006635485
clbN	0/0	100.0	100.0	YP_006635486
clbO	0/0	100.0	100.0	YP_006635487
clbP	0/0	100.0	100.0	YP_006635488
clbQ	0/0	100.0	100.0	YP_006635489
clbS	0/0	100.0	100.0	YP_006635490
clpV/tssH	0/0	100.0	99.0	YP_005226603
cpsACP	0/0	100.0	100.0	YP_002920368
dotU/tssL	0/0	100.0	99.7	YP_005226600
entA	0/0	100.0	100.0	YP_002918380
entB	0/0	100.0	100.0	YP_002918379
entC	0/0	100.0	100.0	YP_002918377
entD	0/0	100.0	100.0	YP_002918366
entE	0/0	100.0	100.0	YP_002918378
entF	0/0	100.0	100.0	YP_002918370
fepA	0/0	100.0	100.0	YP_002918367
fepB	0/0	100.0	100.0	YP_002918376
fepC	0/0	100.0	100.0	YP_002918371
fepD	0/0	100.0	100.0	YP_002918373
fepG	0/0	100.0	100.0	YP_002918372
fes	0/0	100.0	99.9	YP_002918368
fimA	0/0	100.0	100.0	YP_002921137
fimB	0/0	100.0	100.0	YP_002921134
fimC	0/0	100.0	100.0	YP_002921139
fimD	0/0	100.0	100.0	YP_002921140

fimE	0/0	100.0	100.0	YP_002921135
fimF	0/0	100.0	100.0	YP_002921141
fimG	0/0	100.0	100.0	YP_002921142
fimH	0/0	100.0	100.0	YP_002921143
fimI	0/0	100.0	100.0	YP_002921138
fimK	0/0	100.0	100.0	YP_002921144
fyuA	0/0	100.0	100.0	NP_405467
galF	0/0	100.0	100.0	YP_002920369
glf	0/0	100.0	99.9	YP_002920345
gmd	0/0	100.0	100.0	YP_002920358
gnd	0/0	100.0	99.9	YP_002920353
hcp/tssD	0/0	100.0	99.4	YP_005226602
icmF/tssM	0/0	90.0	97.0	YP_005226614
iroD	0/0	100.0	99.0	YP_002920269
iroE	0/0	100.0	100.0	YP_002919453
iroN	0/0	98.9	94.3	YP_002920266
irp1	0/0	100.0	99.0	NP_405471
irp2	0/0	100.0	99.4	NP_405472
iucA	0/0	100.0	100.0	YP_001688094
iucB	0/0	100.0	100.0	YP_001688093
iucC	0/0	100.0	100.0	YP_001688092
iucD	0/0	100.0	100.0	YP_001688091
iutA	0/0	100.0	100.0	YP_001688090
kfoC	0/0	93.5	100.0	YP_002920342
magA/wzy_K1	0/0	100.0	100.0	YP_002920361
manB	0/0	100.0	100.0	YP_002920351
manC	0/0	100.0	100.0	YP_002920352
mrkA	0/0	100.0	100.0	YP_002921128
mrkB	0/0	100.0	100.0	YP_002921127
mrkC	0/0	100.0	100.0	YP_002921126
mrkD	0/0	100.0	100.0	YP_002921125
mrkF	0/0	100.0	100.0	YP_002921124
mrkH	0/0	100.0	100.0	YP_002921121
mrkI	0/0	100.0	99.8	YP_002921122
mrkJ	0/0	100.0	100.0	YP_002921123
rcsA	0/0	100.0	100.0	YP_002920216
rcsB	0/0	100.0	100.0	YP_002920501
rmpA	1-1月	99.8	91.3	YP_001687850
sciN/tssJ	0/0	100.0	100.0	YP_005226619
tssF	0/0	100.0	99.6	YP_005226617
tssG	0/0	100.0	99.7	YP_005226618
ugd	0/0	100.0	100.0	YP_002920350
vasE/tssK	0/0	100.0	99.3	YP_005226599
vipA/tssB	0/0	100.0	99.8	YP_005226597

vipA/tssB	0/0	100.0	93.3	YP_005226597
vipB/tssC	0/0	100.0	98.8	YP_005226598
wbbM	0/0	100.0	100.0	YP_002920346
wbbN	0/0	100.0	100.0	YP_002920344
wbbO	0/0	100.0	100.0	YP_002920343
wcaG	0/0	100.0	99.9	YP_002920357
wcaH	0/0	100.0	100.0	YP_002920356
wcaI	0/0	100.0	100.0	YP_002920355
wcaJ	0/0	100.0	99.9	YP_002920354
wcsT	0/0	100.0	100.0	YP_002920359
wza	0/0	100.0	100.0	YP_002920366
wzb	0/0	100.0	100.0	YP_002920365
wzc	0/0	100.0	100.0	YP_002920364
wzi	0/0	100.0	100.0	YP_002920367
wzm	0/0	100.0	100.0	YP_002920348
wzt	0/0	100.0	100.0	YP_002920347
wzx	0/0	100.0	99.9	YP_002920363
yagZ/ecpA	0/0	99.3	90.1	NP_286010
ybdA	0/0	100.0	100.0	YP_002918375
ybtA	0/0	100.0	99.1	NP_405473
ybtE	0/0	100.0	99.6	NP_405468
ybtP	0/0	100.0	98.5	YP_002920246
ybtQ	0/0	100.0	98.1	YP_002920245
ybtS	0/0	100.0	98.5	NP_405477
ybtT	0/0	100.0	99.6	NP_405469
ybtU	0/0	100.0	99.4	YP_002920250
ybtX	0/0	100.0	98.7	YP_002920244

Product
(acrA) acriflavine resistance protein A [AcrAB (VF0568)] [Klebsiella pneumoniae subsp. pneumoniae NTUH-K2044]
(acrB) acriflavine resistance protein B [AcrAB (VF0568)] [Klebsiella pneumoniae subsp. pneumoniae NTUH-K2044]
(allA) ureidoglycolate hydrolase [Allantion utilization (VF0572)] [Klebsiella pneumoniae subsp. pneumoniae NTUH-K2044]
(allB) allantoinase [Allantion utilization (VF0572)] [Klebsiella pneumoniae subsp. pneumoniae NTUH-K2044]
(allC) allantoate amidohydrolase [Allantion utilization (VF0572)] [Klebsiella pneumoniae subsp. pneumoniae NTUH-K2044]
(allD) ureidoglycolate dehydrogenase [Allantion utilization (VF0572)] [Klebsiella pneumoniae subsp. pneumoniae NTUH-K2044]
(allR) DNA-binding transcriptional repressor AllR [Allantion utilization (VF0572)] [Klebsiella pneumoniae subsp. pneumoniae NTUH-K2044]
(allS) DNA-binding transcriptional activator AllS [Allantion utilization (VF0572)] [Klebsiella pneumoniae subsp. pneumoniae NTUH-K2044]
(clbA) colibactin biosynthesis phosphopantetheinyl transferase ClbA [Colibactin (VF0573)] [Klebsiella pneumoniae subsp. pneumoniae 1084]
(clbB) colibactin hybrid non-ribosomal peptide synthetase/type I polyketide synthase ClbB [Colibactin (VF0573)] [Klebsiella pneumoniae subsp. pneumoniae 1084]
(clbC) colibactin polyketide synthase ClbC [Colibactin (VF0573)] [Klebsiella pneumoniae subsp. pneumoniae 1084]
(clbD) colibactin biosynthesis dehydrogenase ClbD [Colibactin (VF0573)] [Klebsiella pneumoniae subsp. pneumoniae 1084]
(clbE) colibactin biosynthesis aminomalonyl-acyl carrier protein ClbE [Colibactin (VF0573)] [Klebsiella pneumoniae subsp. pneumoniae 1084]
(clbF) colibactin biosynthesis dehydrogenase ClbF [Colibactin (VF0573)] [Klebsiella pneumoniae subsp. pneumoniae 1084]
(clbG) colibactin biosynthesis acyltransferase ClbG [Colibactin (VF0573)] [Klebsiella pneumoniae subsp. pneumoniae 1084]
(clbH) colibactin non-ribosomal peptide synthetase ClbH [Colibactin (VF0573)] [Klebsiella pneumoniae subsp. pneumoniae 1084]
(clbI) colibactin polyketide synthase ClbI [Colibactin (VF0573)] [Klebsiella pneumoniae subsp. pneumoniae 1084]
(clbL) colibactin biosynthesis amidase ClbL [Colibactin (VF0573)] [Klebsiella pneumoniae subsp. pneumoniae 1084]
(clbM) precolibactin export MATE transporter ClbM [Colibactin (VF0573)] [Klebsiella pneumoniae subsp. pneumoniae 1084]
(clbN) colibactin non-ribosomal peptide synthetase ClbN [Colibactin (VF0573)] [Klebsiella pneumoniae subsp. pneumoniae 1084]
(clbO) colibactin polyketide synthase ClbO [Colibactin (VF0573)] [Klebsiella pneumoniae subsp. pneumoniae 1084]
(clbP) precolibactin peptidase ClbP [Colibactin (VF0573)] [Klebsiella pneumoniae subsp. pneumoniae 1084]
(clbQ) colibactin biosynthesis thioesterase ClbQ [Colibactin (VF0573)] [Klebsiella pneumoniae subsp. pneumoniae 1084]
(clbS) colibactin self-protection protein ClbS [Colibactin (VF0573)] [Klebsiella pneumoniae subsp. pneumoniae 1084]
(clpV/tssH) type VI secretion system ATPase TssH [T6SS (VF0569)] [Klebsiella pneumoniae subsp. pneumoniae HS11286]
(cpsACP) phosphatase PAP2 family protein [Capsule (VF0560)] [Klebsiella pneumoniae subsp. pneumoniae NTUH-K2044]
(dotU/tssL) type VI secretion system protein DotU/TssL family [T6SS (VF0569)] [Klebsiella pneumoniae subsp. pneumoniae HS11286]
(entA) 23-dihydroxybenzoate-23-dehydrogenase [Ent (VF0562)] [Klebsiella pneumoniae subsp. pneumoniae NTUH-K2044]
(entB) 23-dihydro-23-dihydroxybenzoate synthetase isochroismatase [Ent (VF0562)] [Klebsiella pneumoniae subsp. pneumoniae NTUH-K2044]
(entC) isochorismate synthase [Ent (VF0562)] [Klebsiella pneumoniae subsp. pneumoniae NTUH-K2044]
(entD) enterochelin synthetase component D [Ent (VF0562)] [Klebsiella pneumoniae subsp. pneumoniae NTUH-K2044]
(entE) enterobactin synthase subunit E [Ent (VF0562)] [Klebsiella pneumoniae subsp. pneumoniae NTUH-K2044]
(entF) enterobactin synthase subunit F [Ent (VF0562)] [Klebsiella pneumoniae subsp. pneumoniae NTUH-K2044]
(fepA) outer membrane receptor FepA [Ent (VF0562)] [Klebsiella pneumoniae subsp. pneumoniae NTUH-K2044]
(fepB) iron-enterobactin transporter periplasmic binding protein [Ent (VF0562)] [Klebsiella pneumoniae subsp. pneumoniae NTUH-K2044]
(fepC) iron-enterobactin transporter ATP-binding protein [Ent (VF0562)] [Klebsiella pneumoniae subsp. pneumoniae NTUH-K2044]
(fepD) iron-enterobactin transporter membrane protein [Ent (VF0562)] [Klebsiella pneumoniae subsp. pneumoniae NTUH-K2044]
(fepG) iron-enterobactin transporter permease [Ent (VF0562)] [Klebsiella pneumoniae subsp. pneumoniae NTUH-K2044]
(fes) enterobactin/ferric enterobactin esterase [Ent (VF0562)] [Klebsiella pneumoniae subsp. pneumoniae NTUH-K2044]
(fimA) type 1 major fimbrial subunit precursor [Type I fimbriae (VF0566)] [Klebsiella pneumoniae subsp. pneumoniae NTUH-K2044]
(fimB) tyrosine recombinase [Type I fimbriae (VF0566)] [Klebsiella pneumoniae subsp. pneumoniae NTUH-K2044]
(fimC) periplasmic chaperone [Type I fimbriae (VF0566)] [Klebsiella pneumoniae subsp. pneumoniae NTUH-K2044]
(fimD) outer membrane usher protein [Type I fimbriae (VF0566)] [Klebsiella pneumoniae subsp. pneumoniae NTUH-K2044]

(fimE) tyrosine recombinase [Type I fimbriae (VF0566)] [Klebsiella pneumoniae subsp. pneumoniae NTUH-K2044]
(fimF) type 1 fimbrial minor component [Type I fimbriae (VF0566)] [Klebsiella pneumoniae subsp. pneumoniae NTUH-K2044]
(fimG) type 1 fimbrial minor component [Type I fimbriae (VF0566)] [Klebsiella pneumoniae subsp. pneumoniae NTUH-K2044]
(fimH) type 1 fimbrial adhesin precursor [Type I fimbriae (VF0566)] [Klebsiella pneumoniae subsp. pneumoniae NTUH-K2044]
(fimI) type 1 pilus biosynthesis fimbrial protein [Type I fimbriae (VF0566)] [Klebsiella pneumoniae subsp. pneumoniae NTUH-K2044]
(fimK) transcriptional regulator [Type I fimbriae (VF0566)] [Klebsiella pneumoniae subsp. pneumoniae NTUH-K2044]
(fyuA) pesticin/yersiniabactin receptor protein [Yersiniabactin (VF0136)] [Yersinia pestis CO92]
(galF) UTP-glucose-1-phosphate uridylyltransferase subunit GalF [Capsule (VF0560)] [Klebsiella pneumoniae subsp. pneumoniae NTUH-K2044]
(glf) UDP-galactopyranose mutase [LPS (VF0561)] [Klebsiella pneumoniae subsp. pneumoniae NTUH-K2044]
(gmd) GDP-D-mannose dehydratase [Capsule (VF0560)] [Klebsiella pneumoniae subsp. pneumoniae NTUH-K2044]
(gnd) 6-phosphogluconate dehydrogenase [Capsule (VF0560)] [Klebsiella pneumoniae subsp. pneumoniae NTUH-K2044]
(hcp/tssD) type VI secretion system protein Hcp family [T6SS (VF0569)] [Klebsiella pneumoniae subsp. pneumoniae HS11286]
(icmF/tssM) type VI secretion protein TssM [T6SS (VF0569)] [Klebsiella pneumoniae subsp. pneumoniae HS11286]
(iroD) siderophore esterase IroD [Sal (VF0563)] [Klebsiella pneumoniae subsp. pneumoniae NTUH-K2044]
(iroE) siderophore esterase IroE [Sal (VF0563)] [Klebsiella pneumoniae subsp. pneumoniae NTUH-K2044]
(iroN) salmochelin receptor IroN [Sal (VF0563)] [Klebsiella pneumoniae subsp. pneumoniae NTUH-K2044]
(irp1) yersiniabactin biosynthetic protein Irp1 [Yersiniabactin (VF0136)] [Yersinia pestis CO92]
(irp2) yersiniabactin biosynthetic protein Irp2 [Yersiniabactin (VF0136)] [Yersinia pestis CO92]
(iucA) aerobactin Synthetase IucA [Aerobactin (VF0565)] [Klebsiella pneumoniae subsp. pneumoniae NTUH-K2044]
(iucB) N-acetyltransferase IucB [Aerobactin (VF0565)] [Klebsiella pneumoniae subsp. pneumoniae NTUH-K2044]
(iucC) aerobactin siderophore biosynthesis protein IucC [Aerobactin (VF0565)] [Klebsiella pneumoniae subsp. pneumoniae NTUH-K2044]
(iucD) lysine 6-monooxygenase IucD [Aerobactin (VF0565)] [Klebsiella pneumoniae subsp. pneumoniae NTUH-K2044]
(iutA) ferric aerobactin receptor IutA [Aerobactin (VF0565)] [Klebsiella pneumoniae subsp. pneumoniae NTUH-K2044]
(kfoC) glycosyltransferase family 2 protein [LPS (VF0561)] [Klebsiella pneumoniae subsp. pneumoniae NTUH-K2044]
(magA/wzy_K1) mucoviscosity-associated protein [Capsule (VF0560)] [Klebsiella pneumoniae subsp. pneumoniae NTUH-K2044]
(manB) phosphomannomutase [Capsule (VF0560)] [Klebsiella pneumoniae subsp. pneumoniae NTUH-K2044]
(manC) mannose-1-phosphate guanylyltransferase [Capsule (VF0560)] [Klebsiella pneumoniae subsp. pneumoniae NTUH-K2044]
(mrkA) type 3 fimbrial major pilin subunit MrkA [Type 3 fimbriae (VF0567)] [Klebsiella pneumoniae subsp. pneumoniae NTUH-K2044]
(mrkB) fimbrial chaperone protein mrkB precursor [Type 3 fimbriae (VF0567)] [Klebsiella pneumoniae subsp. pneumoniae NTUH-K2044]
(mrkC) fimbrial biogenesis outer membrane usher protein mrkC precursor [Type 3 fimbriae (VF0567)] [Klebsiella pneumoniae subsp. pneumoniae NTUH-K2044]
(mrkD) fimbrial adhesin protein precursor MrkD [Type 3 fimbriae (VF0567)] [Klebsiella pneumoniae subsp. pneumoniae NTUH-K2044]
(mrkF) type 3 fimbrial minor pilin subunit MrkF [Type 3 fimbriae (VF0567)] [Klebsiella pneumoniae subsp. pneumoniae NTUH-K2044]
(mrkH) transcriptional activator [Type 3 fimbriae (VF0567)] [Klebsiella pneumoniae subsp. pneumoniae NTUH-K2044]
(mrkI) LuxR family regulatory protein [Type 3 fimbriae (VF0567)] [Klebsiella pneumoniae subsp. pneumoniae NTUH-K2044]
(mrkJ) phosphodiesterase [Type 3 fimbriae (VF0567)] [Klebsiella pneumoniae subsp. pneumoniae NTUH-K2044]
(rcsA) transcriptional activator for ctr capsule biosynthesis [RcsAB (VF0571)] [Klebsiella pneumoniae subsp. pneumoniae NTUH-K2044]
(rcsB) transcriptional regulator RcsB [RcsAB (VF0571)] [Klebsiella pneumoniae subsp. pneumoniae NTUH-K2044]
(rmpA) regulator of mucoid phenotype RmpA [RmpA (VF0570)] [Klebsiella pneumoniae subsp. pneumoniae NTUH-K2044]
(sciN/tssJ) type VI secretion system lipoprotein TssJ [T6SS (VF0569)] [Klebsiella pneumoniae subsp. pneumoniae HS11286]
(tssF) type VI secretion system baseplate subunit TssF [T6SS (VF0569)] [Klebsiella pneumoniae subsp. pneumoniae HS11286]
(tssG) type VI secretion system baseplate subunit TssG [T6SS (VF0569)] [Klebsiella pneumoniae subsp. pneumoniae HS11286]
(ugd) UDP-glucose 6-dehydrogenase [Capsule (VF0560)] [Klebsiella pneumoniae subsp. pneumoniae NTUH-K2044]
(vasE/tssK) type VI secretion system baseplate subunit TssK [T6SS (VF0569)] [Klebsiella pneumoniae subsp. pneumoniae HS11286]
(vipA/tssB) type VI secretion system contractile sheath small subunit VipA [T6SS (VF0569)] [Klebsiella pneumoniae subsp. pneumoniae HS11286]

(vipA/tssB) type VI secretion system contractile sheath small subunit VipA [T6SS (VF0569)] [Klebsiella pneumoniae subsp. pneumoniae HS11286]
(vipB/tssC) type VI secretion system contractile sheath large subunit VipB [T6SS (VF0569)] [Klebsiella pneumoniae subsp. pneumoniae HS11286]
(wbbM) glycosyltransferase [LPS (VF0561)] [Klebsiella pneumoniae subsp. pneumoniae NTUH-K2044]
(wbbN) glycosyltransferase [LPS (VF0561)] [Klebsiella pneumoniae subsp. pneumoniae NTUH-K2044]
(wbbO) glycosyltransferase family 1 protein [LPS (VF0561)] [Klebsiella pneumoniae subsp. pneumoniae NTUH-K2044]
(wcaG) GDP-fucose synthetase [Capsule (VF0560)] [Klebsiella pneumoniae subsp. pneumoniae NTUH-K2044]
(wcaH) GDP-mannose mannosyl hydrolase [Capsule (VF0560)] [Klebsiella pneumoniae subsp. pneumoniae NTUH-K2044]
(wcaI) colanic acid biosynthesis glycosyltransferase WcaI [Capsule (VF0560)] [Klebsiella pneumoniae subsp. pneumoniae NTUH-K2044]
(wcaJ) undecaprenyl-phosphate glucose phosphotransferase [Capsule (VF0560)] [Klebsiella pneumoniae subsp. pneumoniae NTUH-K2044]
(wcsT) galactoside O-acetyltransferase [Capsule (VF0560)] [Klebsiella pneumoniae subsp. pneumoniae NTUH-K2044]
(wza) capsule polysaccharide export protein precursor [Capsule (VF0560)] [Klebsiella pneumoniae subsp. pneumoniae NTUH-K2044]
(wzb) protein tyrosine phosphatase [Capsule (VF0560)] [Klebsiella pneumoniae subsp. pneumoniae NTUH-K2044]
(wzc) inner membrane tyrosine autokinase [Capsule (VF0560)] [Klebsiella pneumoniae subsp. pneumoniae NTUH-K2044]
(wzi) surface assembly of capsule [Capsule (VF0560)] [Klebsiella pneumoniae subsp. pneumoniae NTUH-K2044]
(wzm) lipopolysaccharide O-antigen ABC transport system transmembrane component [LPS (VF0561)] [Klebsiella pneumoniae subsp. pneumoniae NTUH-K2044]
(wzt) lipopolysaccharide O-antigen ABC transport system ATP-binding component [LPS (VF0561)] [Klebsiella pneumoniae subsp. pneumoniae NTUH-K2044]
(wzx) repeat unit exporter [Capsule (VF0560)] [Klebsiella pneumoniae subsp. pneumoniae NTUH-K2044]
(yagZ/ecpA) E. coli common pilus structural subunit EcpA [ECP (VF0404)] [Escherichia coli O157:H7 str. EDL933]
(ybdA) enterobactin exporter EntS [Ent (VF0562)] [Klebsiella pneumoniae subsp. pneumoniae NTUH-K2044]
(ybtA) transcriptional regulator YbtA [Yersiniabactin (VF0136)] [Yersinia pestis CO92]
(ybtE) yersiniabactin siderophore biosynthetic protein [Yersiniabactin (VF0136)] [Yersinia pestis CO92]
(ybtP) yersiniabactin ABC transporter ATP-binding/permease protein YbtP [Ybt (VF0564)] [Klebsiella pneumoniae subsp. pneumoniae NTUH-K2044]
(ybtQ) yersiniabactin ABC transporter ATP-binding/permease protein YbtQ [Ybt (VF0564)] [Klebsiella pneumoniae subsp. pneumoniae NTUH-K2044]
(ybtS) salicylate synthase Irp9 [Yersiniabactin (VF0136)] [Yersinia pestis CO92]
(ybtT) type II thioesterase YbtT [Yersiniabactin (VF0136)] [Yersinia pestis CO92]
(ybtU) yersiniabactin biosynthesis oxidoreductase YbtU [Ybt (VF0564)] [Klebsiella pneumoniae subsp. pneumoniae NTUH-K2044]
(ybtX) yersiniabactin-associated zinc MFS transporter YbtX [Ybt (VF0564)] [Klebsiella pneumoniae subsp. pneumoniae NTUH-K2044]
