# Supplementary material for: First Report of an Extensively Drug-Resistant ST23 Klebsiella pneumoniae of Capsular Serotype K1 Co-Producing CTX-M-15, OXA-48 and ArmA in Spain
Source: Antibiotics (Basel). 2021 Feb 4;10(2):157. doi: 10.3390/antibiotics10020157 (PMC7913926; doi:10.3390/antibiotics10020157)
Supplement: Supplementary file 1 [file antibiotics-10-00157-s001.zip › antibiotics-1092581-supplementary2.pdf]

Gene	Gaps	Coverage	Identity	Accession
aph(3'')-Ib_5	0/0	100	100.0	AF321551
aph(6)-Id_1	0/0	100	100.0	M28829
bla $_{CTX-M-15}$	0/0	100	100.0	AY044436
bla $_{OXA-48}$	0/0	100	100.0	AY236073
bla $_{SHV-190\_1}$	0/0	100	99.9	KP868753
bla $_{TEM-1B\_1}$	0/0	100	100.0	AY458016
dfrA14_5	0/0	100	99.6	DQ388123
fosA6_1	0/0	97	98.3	KU254579
oqxA_1	0/0	100	99.2	EU370913
oqxB_1	0/0	100	98.8	EU370913
qnrB1_1	0/0	100	100.0	DQ351241
sul1_5	0/0	100	99.9	EU780013
sul2_2	0/0	100	100.0	AY034138

Product
aph(3'')-Ib_5
aph(6)-Id_1
Alternate name; UOE-1
blaOXA-48_1
blaSHV-190_1
Alternate name; blaTEM-1
dfrA14_5
fosA6_1
oqxA_1
oqxB_1
qnrB1_1
sul1_5
sul2_2
